# Telomere shortening in laminopathic dilated cardiomyopathy

**DOI:** 10.1038/s41536-026-00462-1

**Published:** 2026-02-10

**Authors:** Alex C. Y. Chang, Gaspard Pardon, Andrew C. H. Chang, Chenguang Wang, Vittavat Termglinchan, Anna Kirillova, Luka Nicin, Foster Birnbaum, Annie Laquerrière, Gisele Bonne, Joseph Wu, Helen M. Blau

**Affiliations:** 1 Department of Cardiology, Ninth People’s Hospital, Shanghai Jiao Tong University School of Medicine, Shanghai, China; 2 Baxter Laboratory for Stem Cell Biology, Department of Microbiology and Immunology, Institute for Stem Cell Biology and Regenerative Medicine, Stanford University School of Medicine, Stanford, CA USA; 3 Stanford Cardiovascular Institute, Stanford University School of Medicine, Stanford, CA USA; 4 Division of Cardiovascular Medicine, Stanford University School of Medicine, Stanford, CA USA; 5 Univ Rouen Normandie, INSERM U1245, Team Epigenetics and Pathophysiology of Neurodevelopmental Disorders and Department of Pathology, Rouen University Hospital, Rouen, France; 6 Centre de Recherche en Myologie, Institut de Myologie, Sorbonne Universités, Paris, France

**Keywords:** Cardiology, Cell biology, Diseases, Genetics, Physiology

## Abstract

Laminopathies are a group of rare disease due to mutations in the *LMNA* gene, which is crucial for nuclear integrity and cellular rigidity. Depending on the mutation, the disease manifests in striated muscles, adipose tissues, nerves, and the heart. Although many laminopathic patients exhibit accelerated aging syndromes, the connection as to why loss of LMNA drives aging remains unknown. Herein, we present evidence that cardiomyocytes from laminopathic heart sections exhibit shortened telomeres. Patient derived hiPSC-CMs we observed *LMNA* mutation results in myocardial enlargement and altered contractility in cardiomyocytes. Further, laminopathic murine cardiomyocytes recapitulates telomere attrition phenotype.

## Introduction

Within cells, the nuclear envelope is responsible for storing genomic material while allowing import and export of biomolecules, and its structure consists of nuclear membranes, nuclear lamina, and nuclear pore complexes. Lamin proteins are the major components of the nuclear lamina residing at the inner nuclear membrane facing chromatin. Laminopathy was first described in the late 1990s and coined in 2000 where mutations of the *LMNA* gene, that encodes lamin A and C, manifests in striated muscles, adipose tissues, nerves, and the heart. Clinically, laminopathies are classified as autosomal dominant Emery-Dreifuss muscular dystrophy (AD-EDMD)^1^, dilated cardiomyopathy with conduction defects (DCM-CD)^2,3^, and limb-girdle muscular dystrophy with cardiac conduction disturbances (LGMD1B)^4^. Cardiac laminopathy results in a wide spectrum of clinical manifestation, including electrical and mechanical alteration of cardiomyocytes contractility, cardiac fibrosis and arrythmias, leading towards life-threatening complication including sudden cardiac death and end-stage heart failure^2,5^. Besides heart transplants, currently there is no cure for this devastating disease.

Telomeres, composed of TTAGGG repeats located at the ends of chromosomes, maintain genomic stability and their shortening is a hallmark of aging^6^. It has been shown that cardiomyocytes remain largely non-proliferative post-birth^7^ and healthy myocardial telomere lengths remain relatively stable^8^. Previously we have demonstrated that telomere shortening occurs independent of proliferation in cardiomyopathies driven by sarcomeric mutations^9–11^ and it is now accepted that non-proliferative cells can also enter ‘senescence-like’ states^12^. Moreover, it has been shown that telomere localization is dependent on nuclear geometry^13^ Thus, we wondered whether perturbation to nuclear integrity due to *LMNA* mutation can result in accelerated telomere shortening due to increased contractile stress on the nucleus^14^. Here we show in laminopathic patient cardiac sections that loss of nuclear integrity correlates in telomere shortening. Further, using isogenic human induced pluripotent stem cell (hiPSC) and hiPSC-derived cardiomyocytes (hiPSC-CM) with or without mutation on one or two alleles, we demonstrate that telomere lengths are governed by lamin A/C levels and is cell type specific. We further observed that *LMNA* mutation results in increased cardiomyocyte surface area and increased contractile force.

## Results

### Laminopathic cardiomyocytes exhibit telomere shortening

**Fig. 1:**
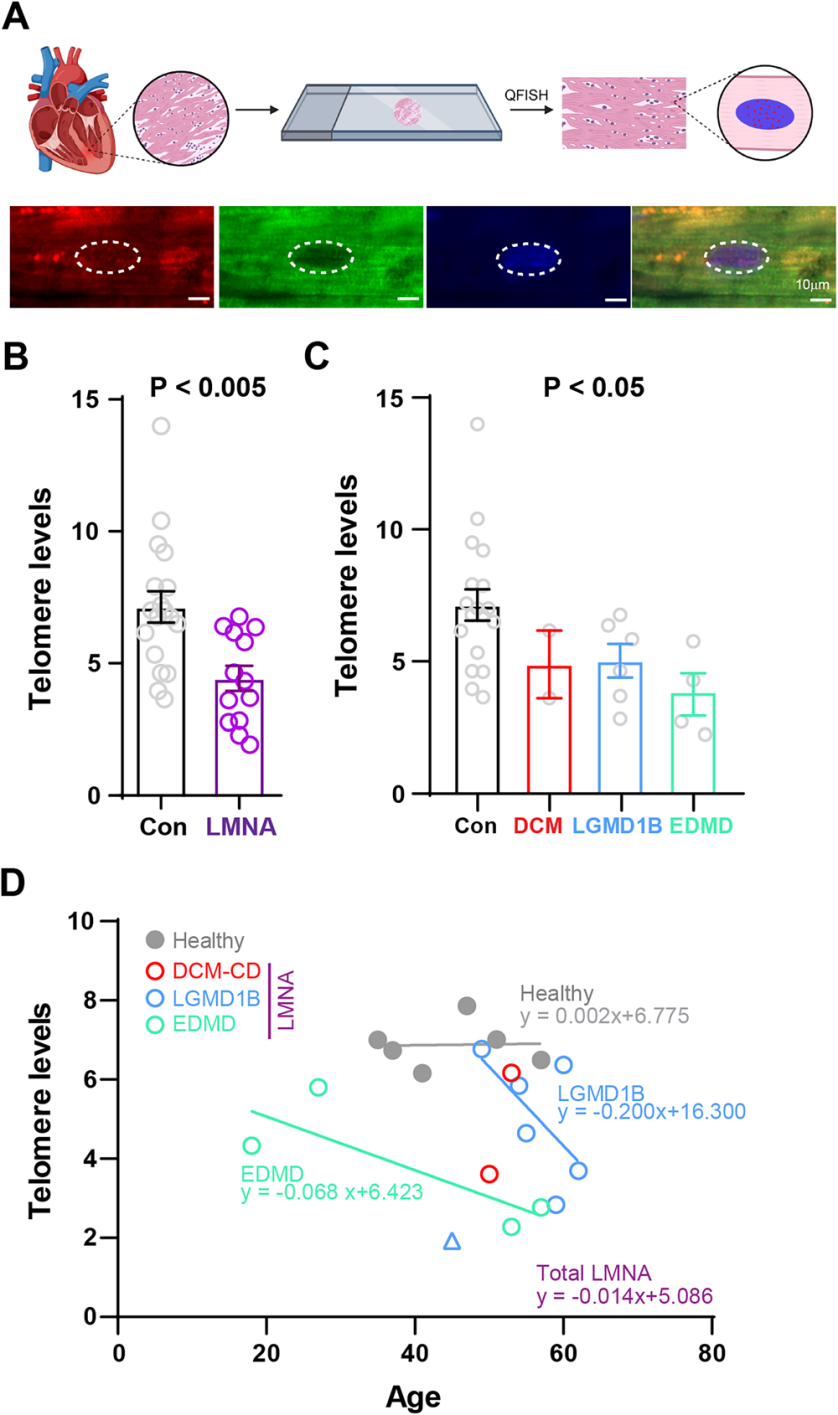
Cardiomyocytes from patients suffering laminopathies exhibit shortened telomeres. **A** Paraffin-embedded cardiac samples of laminopathy patients (*N* = 13 patients; *n* = 130–540 nuclei per patient tissue), control patients (healthy: *N* = 6 patients; *n* = 91-186 nuclei per patient tissue, and transplanted hearts: N = 12; *n* = 133–887 nuclei per patient tissue) were used for myocardial telomere Q-FISH quantification. Telomere intensities (a.u.) were scored in a blinded fashion within five to six regions of interest in two nonconsecutive sections. Created in BioRender. Wang, C. (2025) https://BioRender.com/8hmmn43. **B** Telomere levels per patient is or (**C**) per genotype are plotted. Data represent mean ± SEM. Student’s t-test for (**B**) and one-way ANOVA tests for (**C**) were used to calculate significance. **D** Average telomere levels and age of laminopathy patients and healthy controls are plotted and best-fit linear curves are shown. Extremely low telomere LGMD1B sample (blue triangle) was considered an outlier and was not included for determining best-fit linear curve.

To determine if mutation in *LMNA* gene would result in telomere shortening, we performed quantitative FISH (Q-FISH) and measured telomere fluorescence intensity per nucleus in cardiomyocytes (CMs) expressing the CM-specific marker Troponin-T (Fig. 1A). Cardiac sections from laminopathic patients who received heart transplant between 1997 and 2012 (Table 1) as well as healthy controls (Supplementary Table S1) were used. Hematoxylin and eosin staining showed presence of cardiac remodeling in all *LMNA* samples (Supplemental Figs. S1 & S2). Control biopsies of the grafted hearts were also included for telomere analysis. In accordance with our previous findings in hypertrophic and dilated cardiomyopathic cardiomyocytes^11^, the telomere levels of Troponin-T+ CMs in *LMNA* hearts were significantly reduced by 38% compared with healthy controls (*LMNA*, *n* = 13, 4.43 ± 0.48; control, *n* = 18, 7.13 ± 0.60; Fig. 1B). The trend is consistent when results were grouped based on clinical phenotype (DCM-CD, *n* = 2, 4.89 ± 1.28; EDMD, *n* = 4, 3.80 ± 0.80; LGMD1B, *n* = 6, 5.03 ± 0.64; Fig. 1C). Next, we examined the correlation between telomere level and age. Given the ages of the healthy donor hearts were not disclosed, we only compared telomere levels of *LMNA* and healthy controls. Healthy cardiomyocytes showed no telomere shortening across age which is in keep with previous observation^15^; LGMD1B patients exhibited faster telomere loss compared to EDMD patients (Fig. 1D). Together, these results show that loss of *LMNA* results in telomere loss in cardiomyocytes.

**Table 1:** Laminopathy patient cardiac samples used for telomere quantification

Phenotype	Gender	Age of transplant	Molecular Anomaly	Expected Protein Consequences	Onset of Cardiac Phenotype	Clinical Manifestations
DCM-CD	F	50	*LMNA:* c.28dup; p.Thr10AsnfsX31	haplo *LMNA*	34 yo: VT	AVB-I, non sustained and sustained VT requiring ICD at 45 yo, AF, DCM
DCM-CD	F	53	*LMNA:* c.28dup; p.Thr10AsnfsX31	haplo *LMNA*	Unknown	AF + DCM
EDMD	M	18	*LMNA:* c1135C>T; p.Leu379Phe	missense *LMNA*	12 yo: PVC + non sustained VT	myopathy with joint contractures + AF + incomplete LBBB + PVC + sustained VTDCM
EDMD	M	27	EMD: c.600-313del ; p.Trp200CysfsX5	absence of emerin	24 yo: ventricular fibrillation, sudden death	myopathy with joint contractures, sudden death, ventricular fibrillation requiring ICD at 24 yo, bifascicular block, DCM,
EDMD	M	53	*LMNA:*c.16 C > T; p.Gln6X	haplo *LMNA*	36 yo: AF	myopathy with joint contractures, AF, non sustained and sustained VT requiring ICD at 52 yo, DCM
EDMD	M	57	*LMNA:*c.16 C > T; p.Gln6X	haplo *LMNA*	23 yo: AVB-I, PVC	myopathy with joint contractures, AF, AVB-III requiring PM, on sustained and sustained VT requiring ICD at 47 yo, DCM
LGMD1B	F	45	*LMNA:* c.746 G > C; p.Arg249Pro	missense *LMNA*	40 yo: AF	proximal myopathy + AVB-III requiring PM at 42, DCM
LGMD1B	F	49	*LMNA:* c.28dup; p.Thr10AsnfsX31	haplo *LMNA*	Unknown	proximal myopathy AF + AVB-I + VT + DCM
LGMD1B	F	54	*LMNA:* c.28dup; p.Thr10AsnfsX31	haplo *LMNA*	46 yo: PAC + AVB-I + Luciani-Wenckebach periods requiring PM	proximal myopathy + AF + DCM
LGMD1B	M	55	*LMNA:* c.28dup; p.Thr10AsnfsX31	haplo *LMNA*	Unknown	proximal myopathy + AF + AVB-I + DCM
LGMD1B	M	59	*LMNA:* c.28dup; p.Thr10AsnfsX31	haplo *LMNA*	Unknown	proximal myopathy + AF + AVB-III requiring PM, VT requiring ICD at 57 yo + DCM
LGMD1B	F	60	*LMNA:* c.28dup; p.Thr10AsnfsX31	haplo *LMNA*	38 yo: AF	proximal myopathy + AF + AVB-I and AVB-II requiring PM, sustained VT requiring ICD at 54 yo, DCM
LGMD1B	F	62	*LMNA:* c.28dup; p.Thr10AsnfsX31	haplo *LMNA*	Unknown	proximal myopathy + AF + DCM

### *LMNA* copy number plays a role in hiPSC and hiPSC-CM telomere length homeostasis

**Fig. 2:**
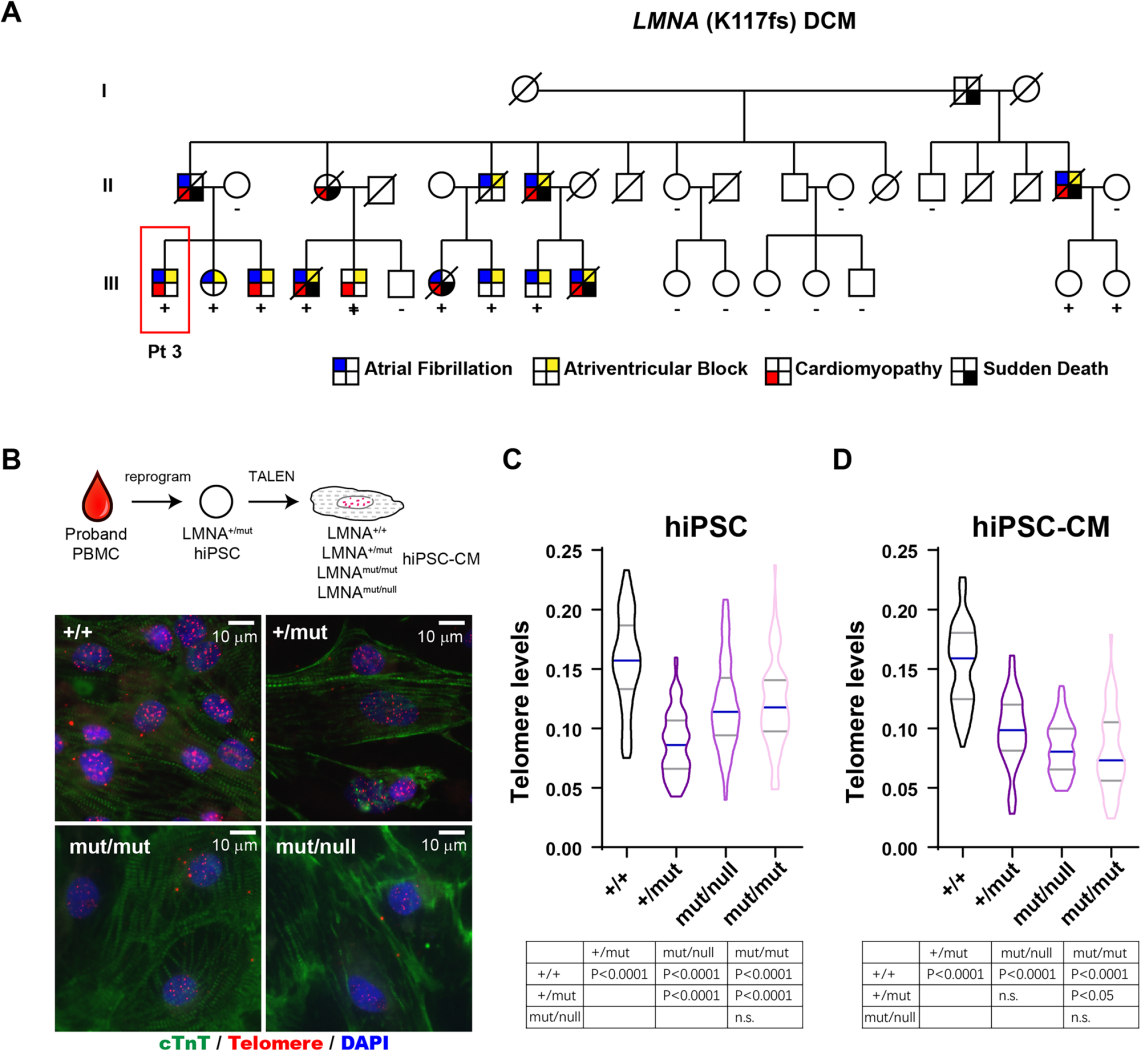
*LMNA* (K117fs) hiPSC and hiPSC-CMs exhibit shortened telomeres. **A** Pt3, part of a three-generation family that had 15 affected members exhibiting autosomal dominant cardiac disease, used for the generation of hiPSC. Square, males; circles, females; slash, deceased. Pedigree originally reported in Heart Rhythm 2009 May; 6(5): 707–710. **B** Generation of hiPSC lines and subsequent hiPSC-CM differentiation. Representative micrographs of *LMNA* + / + , +/mut, mut/mut, and mut/null hiPSC-CMs stained with cardiac troponin cTnT (green), TelC telomeres (red), and nuclei (DAPI) are shown. **C** Telomere levels of hiPSC and (**D**) hiPSC-CMs were quantified. Data are shown as violin plots where blue median and gray quartiles are shown. One-way ANOVA tests by comparing the mean of each group with the mean of every other group, followed by Holm–Sídák multiple comparison test were used.

To study the effect of *LMNA* copy number and its relationship with telomere length homeostasis, we generated human induced pluripotent stem cells from a *LMNA*^+/mut^ patient with a frameshift mutations in exon 1 that results in expression of a truncated protein (Fig. 2A)^16^. Using TALEN, isogenic lines were generated^17,18^. Under aberrant nuclear integrity^19^, we first asked if *LMNA* copy number changes (2 copies, *LMNA*^+/+^; 1 copy, *LMNA*^+/mut^; 0 copy *LMNA*^mut/mut^ and *LMNA*^mut/null^) can affect telomere levels in both hiPSC and hiPSC-CMs (Fig. 2B). In hiPSCs, restoration of *LMNA*^+/+^ copy number lengthened telomeres compared to *LMNA*^+/mut^ cells (Fig. 2C). Interestingly, loss of both *LMNA* copies (*LMNA*^mut/mut^ and *LMNA*^mut/null^) slightly increased telomere levels in hiPSCs compared to *LMNA*^+/mut^, albeit not to the extent of *LMNA*^+/+^ cells (Fig. 2C). However, after differentiation into hiPSC-CMs, both *LMNA*^mut/mut^ and *LMNA*^mut/null^ cardiomyocytes exhibited a further telomere loss compared to *LMNA*^+/mut^ (Fig. 2D). Together, these data support the idea that telomere length homeostasis is regulated by the nuclear envelope integrity, which is determined by *LMNA* expression and the mutational status.

### *LMNA*^+/mut^ results in increased myocardial surface area and increased contractile force

**Fig. 3:**
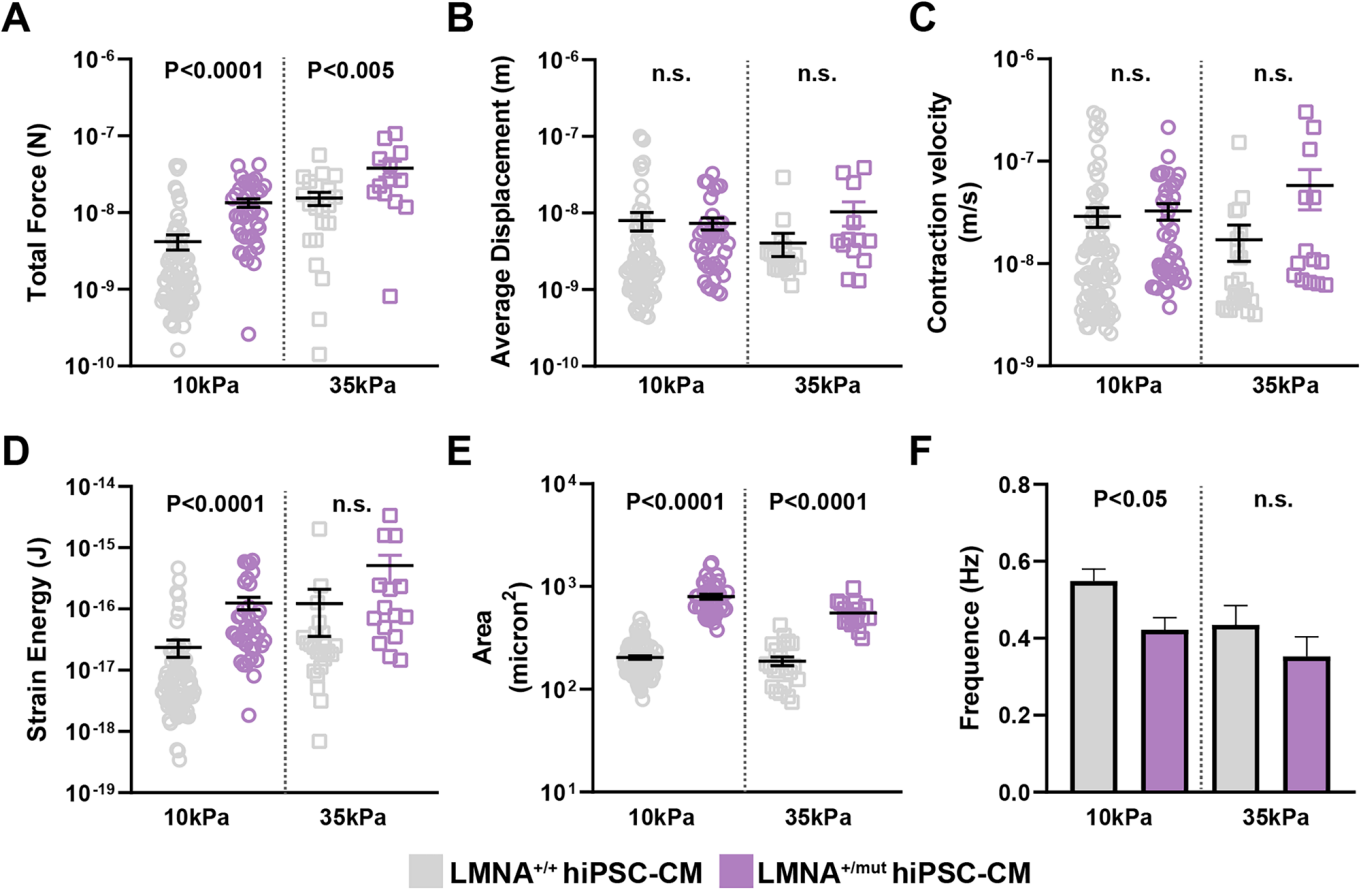
*LMNA*^+/mut^ hiPSC-CMs exhibit altered force generation measured by traction force microscopy. Contractile assessment of *LMNA*^+/mut^ and isogenic control *LMNA*^+/+^ hiPSC-CMs using traction force microscopy was performed on 10 kPa or 35 kPa hydrogels. (**A**) Force, (**B**) Displacement, (**C**) Contraction velocity, (**D**) Strain, (**E**) Area, and (**F**) beat rate of single *LMNA*^+/+^ and *LMNA*^+/mut^ hiPSC-CMs subjected to 10 kPa and 35 kPa hydrogels under 1 Hz electrical pacing were measured (*n* = 3 independent experiments, 17–23 cells were analyzed). Data represent mean ± SEM. Student’s t-tests were used to calculate significance.

To evaluate if changes in *LMNA* alters a cardiomyocyte’s ability to generate force when challenged with a fibrotic environment^10^, we subjected *LMNA*^+/mut^ and *LMNA*^+/+^ hiPSC-CMs to traction force microscopy under electrical pacing at 1 Hz^20^. Single hiPSC-CMs were seeded on micropatterned hydrogel substrates to provide them a more physiological elongated shape and the hydrogel substrate stiffness was controlled to mimic healthy (10 kPa) and fibrotic (35 kPa) conditions. Interestingly, we observed that *LMNA*^+/mut^ hiPSC-CMs displayed increased cell size (Fig. 3A) compared to *LMNA*^+/+^ hiPSC-CMs independently of substrate stiffness. Consequently, we measured that *LMNA*^+/mut^ hiPSC-CMs produced overall more force compared to *LMNA*^+/+^ hiPSC-CMs (Fig. 3B), which was somewhat unexpected. However, while *LMNA*^+/mut^ hiPSC-CMs also produced more strain energy under 10 kPa condition, (Fig. 3C); this was not the case when hiPSC-CMs were cultured under 35 kPa condition, which reveal an increased sensitivity to fibrotic stiffening in *LMNA*^+/mut^ hiPSC-CMs. Since there was no difference in terms of average displacement (i.e., average hydrogel strain deformation) (Fig. 3D), the differences in terms of total force and strain energy between *LMNA*^+/+^ and *LMNA*^+/mut^ can be attributed to the differences in cell size. Interestingly, cell size has been reported to correlate with nuclear size and inversely to lamin expression^21^. *LMNA* did not alter the contraction velocity (Fig. 3D, E) which suggests intact sarcomeric structure. However, under controlled electrical pacing conditions, we still observed small differences in average beating frequency (Fig. 3F), revealing potential arrhythmic contractions and deficient calcium handling capabilities. Together, these results suggest that expression of the *LMNA* truncated variant increases myocardial cell size and results in substrate-stiffness sensitive contractile force, strain energy generation, as well as in loss of beating rhythmicity.

### Cardiomyocytes from h*LMNA*-c.C1824T knockin mice also exhibit telomere shortening

**Fig. 4:**
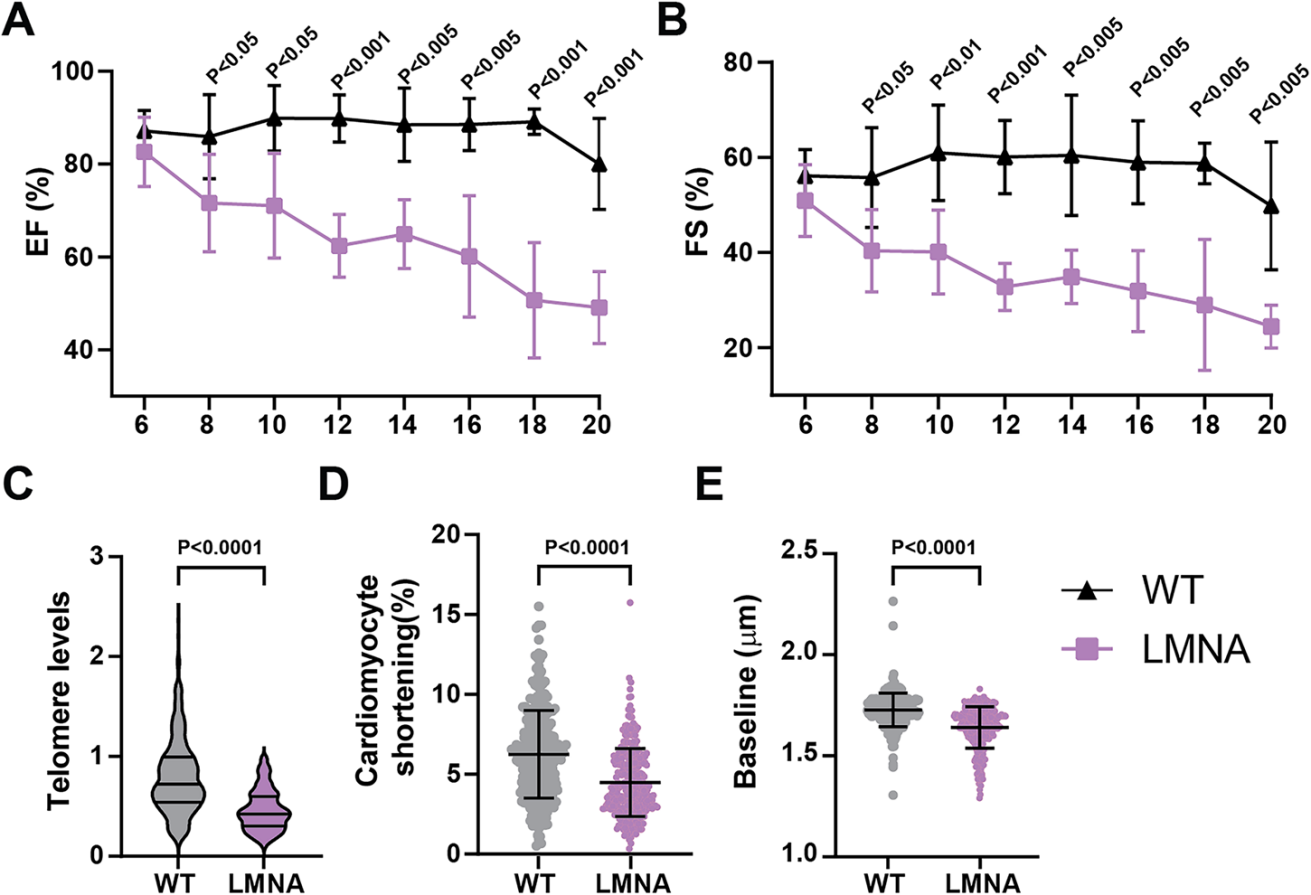
C57BL/6 J h*LMNA*-c.C1824T heterozygous animals exhibit cardiac dysfunction and myocardial telomere shortening. Cardiac function evaluated by echocardiography. **A** Left ventricular ejection fraction (EF %) and (**B**) fractional shortening (FS %) at 6, 8, 10, 12, 14, 16, 18, and 20 weeks of age are shown. (*n* = 5 animals each). **C** Telomere intensities (a.u.) were scored in a blinded fashion within five regions of interest in two nonconsecutive sections per animal, five animals per group with a total of *n* = 126–138 nuclei per mouse scored. Quantification of (**D**) cardiomyocyte shortening and (**E**) sarcomeric baseline of Langendorff isolated cardiomyocytes are shown. Data are represented as mean ± SEM. Student’s *t*-tests were used to calculate significance.

To evaluate whether *LMNA* mutation or loss of expression can drive myocardial telomere shortening, we used heterozygous h*LMNA*-c.C1824T knockin mice to evaluate. Echocardiography assessment confirms an early onset of dilated cardiomyopathy in heterozygous h*LMNA*-c.C1824T knockin animals marked by decreased left ventricular ejection fraction (LVEF %) as well as fraction shortening (FS %) (Fig. 4A, B). In presence of h*LMNA*-c.C1824T mutant, myocardial telomere levels were significantly decreased in *LMNA* mice compared to controls (Fig. 4C). In Langendorff isolated cardiomyocytes, *LMNA* cardiomyocytes exhibit a significant decrease in cardiomyocyte shortening as well as sarcomeric baseline distance (Fig. 4D, E), indicative of contractile dysfunction. Together, these results suggest that *LMNA* mutation drives myocardial telomere attrition in heterozygous h*LMNA*-c.C1824T knockin mice.

## Discussion

Here we demonstrate that laminopathic cardiomyocytes exhibit telomere loss and this phenomenon is recapitulated in patient hiPSCs. We observed that cardiomyocytes from patients classified with LGMD1B exhibit faster telomere shortening compared to EDMD patients while healthy controls exhibit very little telomere loss over chronological aging (Fig. 1C). Second, using TALENs^17,18^, we interrogated the relationship between *LMNA* copy number and telomere homeostasis. Using the four hiPSC lines, isogenic wildtype (*LMNA*^+/+^), patient-derived (*LMNA*^+/mut^), homozygous mutant (*LMNA*^mut/mut^) and knockout (*LMNA*^mut/null^), we demonstrate that telomere homeostasis is *LMNA* dependent and also cell-type specific. Telomeres are shorter in all *LMNA* mutated hiPSC lines compared to *LMNA*^+/+^ hiPSCs. Among the three *LMNA* mutational type, the homozygous *LMNA*^+/mut^ mutation appeared to provides the least favorable nuclear integrity for telomere homeostasis in hiPSCs. However, upon differentiation into hiPSC-CMs, the high contractile demand in cardiomyocytes mostly impacted telomere level in *LMNA*^mut/null^ and *LMNA*^mut/mut^, resulting in shorter telomeres in these lines compared to *LMNA*^+/mut^ and control *LMNA*^+/+^ hiPSC-CMs. We also show on single cardiomyocyte level that *LMNA*^+/mut^ hiPSC-CMs increase contractile force compared to *LMNA*^+/+^ hiPSC-CMs, an effect that appeared largely due to increased cell size. Myocardial telomere attrition was further validated in the h*LMNA*-c.C1824T heterozygous knockin mice. Overall, these results suggest that *LMNA* mutations alter telomere homeostasis and this phenomenon is cell-type and mutation-type specific, thereby correlating with the composition of the nuclear envelope and nuclear mechanotransduction. *LMNA* expression level has indeed been shown to affect the binding of the inner membrane protein emerin^14^ and of the structural Nesprins proteins^22^.

Compared to our previous studies where sarcomeric mutations drive telomere shortening in genetic DCM and HCM^11^ and that contraction of diseased cardiomyocytes promote telomere shortening^10^, it is clear from our results that disruption to *LMNA* expression, by various mutations, results in telomere attrition. Further, depending on mutation severity, the rate of telomere loss in laminopathic cardiomyocytes also differ. Although mice with extended telomeres exhibit less metabolic aging and have longer lifespans^23^, it remains elusive how telomere lengths are established and maintained. It has been demonstrated that regardless of starting cells, reprogramming resets the telomere length^24^ and this memory is kept in chimera mice derived from long telomere mESCs^25^. Furthermore, long telomeres, provided by C57BL/6 J mouse background, can override short telomeres, in CAST/Ei or SPRET/Ei background, and establish new telomere set points^26^ and it seems that telomere set points can be inherited^27^. These observations suggest that nuclear integrity, determined by composition of lamins, may determine telomere lengths in cells.

Second, it has been shown that telomeres are captured by the TERB1-TERB2-MAJIN complex and anchored onto nuclear envelope via SUN1 that regulates chromosomal rearrangements during meiosis^28^. Evidence from the Hockemeyer lab has demonstrated that TPP1 and POT1, two telomere binding proteins part of the shelterin complex, are required for telomere length homeostasis^29–31^ and TPP1 phosphorylation has been shown to initiate telomerase recruitment^32^. Whether these signaling pathways are actively present in non-dividing cardiomyocytes and whether these pathways are affected by *LMNA* disruption in the nuclear membrane integrity and nuclear mechanotransduction remains to be explored.

Third, unlike the heterozygous h*LMNA*-c.C1824T knockin mice, *LMNA*^+/mut^ did not exhibit defective contractile force production, which raises the question of how do laminopathic cardiomyopathies develop? The C1824T mutation results in the production of progerin protein that is responsible for Hutchinson-Gilford progeria syndrome (HGPS), a rare genetic disorder mainly characterized by accelerated aging in children. Progerin, which lacks 50 amino acids on its carboxyl terminus, remain farnesylated at the cysteine of its CAAX sequence and prevents ZMPSTE24 protease cleavage to release it from the nuclear membrane^33–35^. To note, C1824T knockin mice^36^ and pigs^37^ have been shown to exhibit cardiac hypertrophy and diastolic dysfunction; whereas, *LMNA* deficient^38^ or myocardial *LMNA* knockout mice^39^ exhibit systolic dysfunction. It is known that isoflurane may suppress systolic function thus the systolic dysfunction we observed in our C1824T knockin animals warrant for further investigation of this new animal model. Transcriptionally, our group has previously demonstrated that *LMNA* loss can increase chromatin open state and non-sense mediated decay in laminopathic cardiomyocytes^19^. Enzymatically, it has been suggested that the leakiness of laminopathic nuclei can result in infiltration of matrix-metalloprotease-2^40^. Moreover, overexpression of a dominant negative SUN1 protein was able to prevent laminopathic cardiomyopathy in mice^39^. However, whether all *LMNA* mutations can drive cardiac phenotype directly or compounded by endothelial dysfunction complications^41^ remains to be elucidated. To note, limited by tissue availability, we were unable to perform traditional telomeric repeat amplification protocol (TRAP) or RT-qPCR methods. The number of *LMNA* mutations examined are limited and further validations are warranted. In this study, we measured telomere signal using QFISH which captures relative telomeric length, but like all probe-based assays, are less sensitive to really short telomeres.

Together, this work provides evidence of accelerate telomere loss in human and murine laminopathic cardiomyocytes. We show that the level of telomere shortening may be correlated with disease severity. Using hiPSC and hiPSC-CMs as model, we demonstrate that truncated *LMNA* acts as a dominant negative in the pluripotent state, while the lack of full-length *LMNA* contributes to telomere attrition in differentiated cardiomyocytes. *LMNA*^+/mut^ hiPSC-CMs exhibited larger cell size and produced more force when challenged with healthy and fibrotic-mimicking hydrogels, suggestive of altered cellular structure. Further, myocardial telomere attrition was further confirmed in the h*LMNA*-c.C1824T knockin mice. Our results provide the necessary tools in supporting the possibility of targeting telomeric ends in the treatment of laminopathic cardiomyopathies.

## Methods

### Human cardiac samples

The use of human samples was reviewed and approved by the Stanford Institutional Review Board (no. 13465). Deidentified laminopathic and engrafted heart sections were obtained from Normandie University, INSERM. Control hearts were isolated < 24 h post mortem from deidentified male patients who died of noncardiac disease at University of British Columbia. All tissue samples were formalin fixed, paraffin embedded, and 4 μm sections placed on ChemMate slides (Fisher Scientific) were used for telomere Q-FISH staining and telomere signal was quantified using Telometer as previously described in ref. ^11^. Histology was evaluated using hematoxylin and eosin staining (Fisher Scientific) per manufacturer’s instructions.

### hiPSC-CM differentiation

All protocols using hiPSC were reviewed and approved by the Stanford Stem Cell Research Oversight committee (#602) as well as the Ethics Review committee at Ninth People’s Hospital, Shanghai Jiao.

Tong University School of Medicine (2018-207-K32). The *LMNA* patient hiPSC line was generated part of the Stanford Cardiovascular Institute Biobank where subsequent isogenic lines were generated as previously described^17,18^. hiPSC maintenance and hiPSC-CM differentiation were performed using the 2D matrigel protocol and traction force microscopy was used to evaluate contractile function as previously described^10,11^.

### Animals

*LMNA* animals, C57BL/6 J h*LMNA*-c.C1824T knockin mice (Strain NO. T059801), were acquired from GemPharmatech (Nanjing, China). Pathogenic humanized *LMNA* point mutation at exon 11 (c.1824C>T [p.G608G]) was introduced via CRISPR-Cas9 construct according to supplier’s website (https://en.gempharmatech.com/product/details100035_4040548.html). Lethality was observed in homozygous C1824T animals; heterozygous animals were used in function and histological studies.

Animals were kept in an SPF barrier environment with a 12 h light/dark cycle and were provided with sufficient food and water. All animal studies complied with the Declaration of Helsinki, NIH guidelines, and were approved by the Animal Experiment Ethics Committee of Shanghai Ninth People’s Hospital (SH9H-2021-TK500-1). Mice were anaesthetized with intraperitoneal injection of 1.2% 2,2,2-tribromoethanol solution (0.2 mL/10 g body weight), monitoring respiratory and heart rates, and euthanized by CO2 overdose. Visual Sonics Vevo 3100 system (Visual Sonics, FUJIFILM) was used to perform echocardiographic assessment. During echocardiography, mice were anaesthetized with 2% isoflurane. Systolic functions were measured at the midventricular long-axis using M-mode scanning while maintaining the heart rate at the range of 425–475 beats per minute. 4 μm sections were used for telomere Q-FISH staining and telomere signal was quantified using Telometer as previously described in ref. ^11^. Murine cardiomyocytes were isolated using the Langendorff method as previously described in ref. ^9^ and an IonOptix HTC System (IonOptix) was used for function assessment. Briefly, Langendorff isolated AMCMs were seeded onto laminin (Sigma)-coated imaging dishes in DMEM supplemented with 10% FBS and incubated for 30 min at 37 °C. The cells were field stimulated (10 V) at a frequency of 1 Hz, and the changes of sarcomere length were simultaneously recorded and calculated.

### Statistics

Statistical differences were analyzed using one-way ANOVA followed by Holm-Sidak’s multiple comparison test or by Student’s t-test as indicated. Image capture and quantification analyses were performed in a double-blinded fashion to avoid bias. All data are shown as the mean ± SEM. Significant differences were determined as *p* < 0.05.

## Supplementary information

Supplementary Information

## Data Availability

All data are available in the main text or the supplementary materials. The raw datasets used and/or analyzed during the current study are available from the corresponding author.
