# Supplementary material for: Telomere shortening in laminopathic dilated cardiomyopathy
Source: NPJ Regen Med. 2026 Feb 10;11:16. doi: 10.1038/s41536-026-00462-1 (PMC13044268; doi:10.1038/s41536-026-00462-1)
Supplement: Supplementary file 1 — Supplementary Information [file 41536_2026_462_MOESM1_ESM.pdf]

Supplementary Table S1. Healthy cardiac samples used for telomere quantification.

Phenotype	Gender	Age	Cause of Death
**Healthy**	F	37	Ruptured cerebrovascular aneurysm, subarachnoid hemorrhage
**Healthy**	M	35	Self-inflicted gunshot wound to head
**Healthy**	M	41	Intraventricular (brain) hemorrhage
**Healthy**	F	51	Choking on food leading to asphixia
**Healthy**	M	47	Benign brain tumor compressing brain circulation, acute EtOH
**Healthy**	F	57	Ruptured cerebrovascular aneurysm, subarachnoid hemorrhage

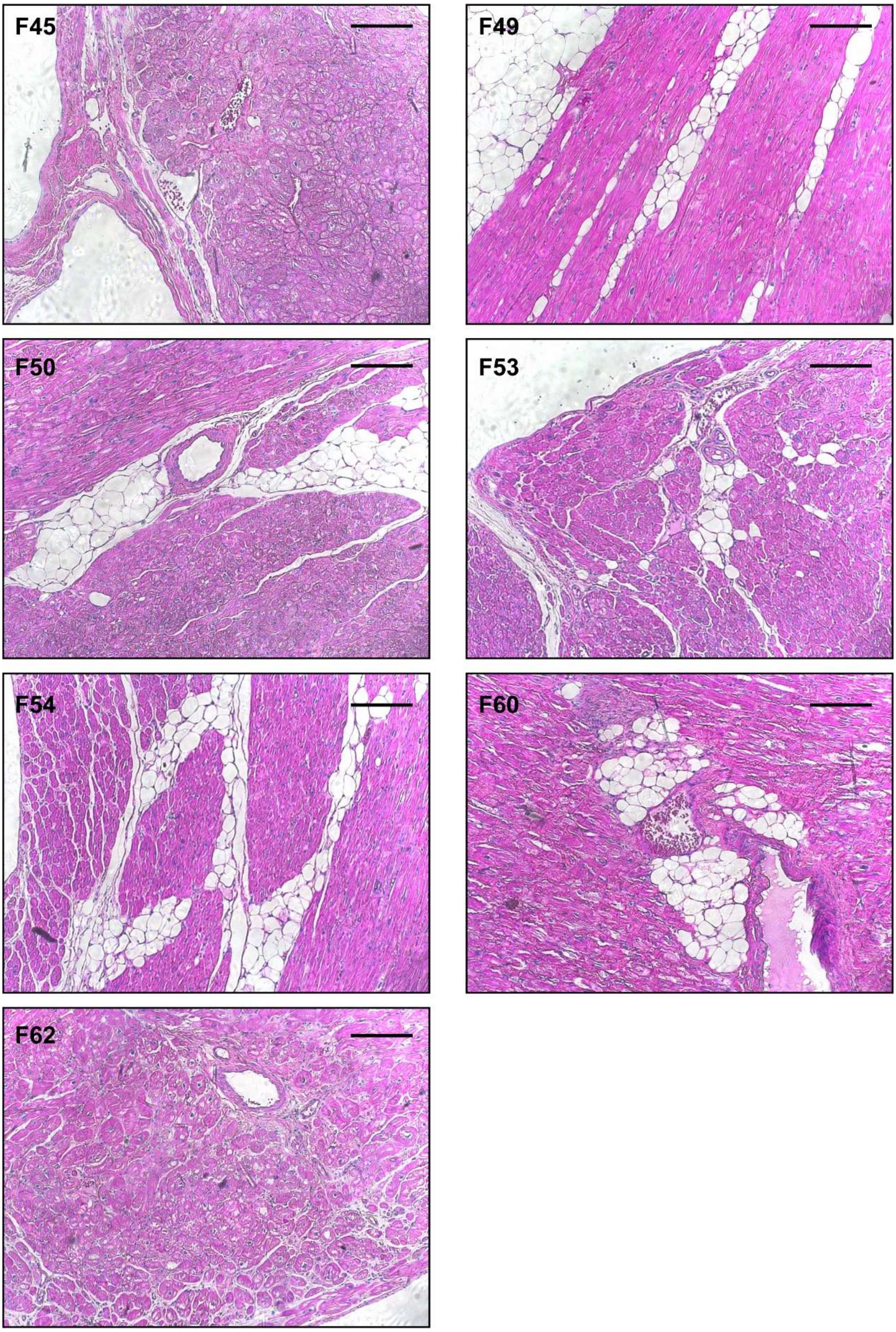

Supplemental Figure S1. Hematoxylin and Eosin staining of female LMNA cardiac sections. Scale bars, 100µm.

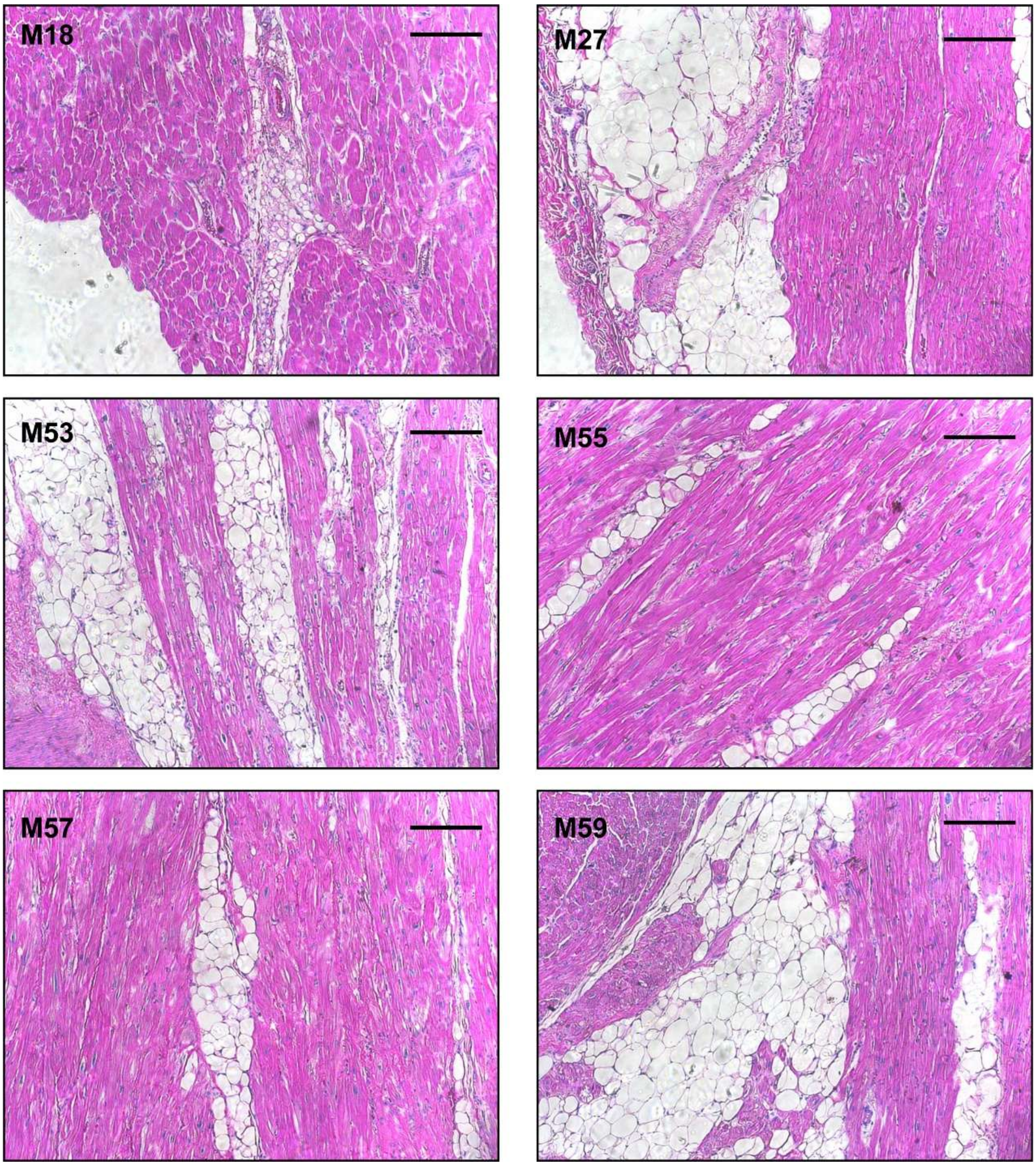

Supplemental Figure S2. Hematoxylin and Eosin staining of male LMNA cardiac sections. Scale bars, 100μm.
